# Editorial: Targeted Immunotherapy for Cancer

**DOI:** 10.3389/fphar.2022.894681

**Published:** 2022-04-12

**Authors:** Williams V. Williams

**Affiliations:** BriaCell Therapeutics Corp., Philadelphia, PA, United States

**Keywords:** Cancer, Immunotherapy, Targeted immunotherapy, Immune modulation—immunological terms, targeted (selective) treatment, Immune checkpoint inhibitor

Targeted immunotherapy for cancer is a field that is on fire with new innovations and notable successes. In this issue numerous papers were contributed expounding on the successes and even more on novel approaches or key insights that may be pivotal in further therapeutic advances.

Several papers were submitted evaluating the ever-expanding literature on the use of immune checkpoint inhibitors (ICIs) in cancer. The bibliometric study of ICI use by Sun et al. includes a description of the historical evolution of ICI use (Sun et al.). They discuss the trends in research into immune checkpoint blockade by anti-PD1/PDL1 antibodies in cancer immunotherapy. Their use of a direct citation network of randomized controlled trials indicated the development of these therapies was transformational for the treatment of many cancers. This paper provides a good historical perspective within which the others evaluating ICIs can be placed. Xue et al. evaluate the association between the efficacy of ICIs and sex (Xue et al.). Their meta-analysis of 12,675 non-small cell lung cancer (NSCLC) patients revealed that ICIs significantly improved overall survival and progression free survival in males and females with no statistical difference between the sexes. However, they also noted that immunotherapy for NSCLC patients had more treatment-emergent adverse events compared with chemotherapy, with insufficient data to compare the sexes for anuy potential differences in the frequency of adverse events. Feng et al. performed a meta-analysis of seven studies of PD-1/PD-L1 and CTLA-4 inhibitor combination therapy (Feng et al.). They found that combination therapy has longer progression-free survival (PFS), overall survival (OS), and better objective response rate (ORR) than other treatments for cancer patients. They also noted that PFS in patients with malignant tumors is positively correlated with PD-L1 expression.

These studies document the tremendous impact ICIs have had already in cancer treatment. Looking forward, Huang et al. evaluate the current landscape and future projections for the use of immune checkpoint inhibitors in the first-line setting (Huang et al.). Looking predominately at NSCLC, they review the history of the developments in cancer immunotherapy, summarize the mechanism of action and based on the results of the recent first-line trials, propose a potential first-line immunotherapeutic strategy for the treatment of the patients with NSCLC. Similarly forward looking, Varayathu et al. evaluated combination strategies to augment ICI efficacy and the implications for translational research (Varayathu et al.). Combinations evaluated included conventional chemotherapy drugs, metronomic chemotherapy, thalidomide and its derivatives, epigenetic therapy, targeted therapy, inhibitors of DNA damage repair, other small molecule inhibitors, anti-tumor antibodies, hormonal therapy, multiple checkpoint Inhibitors, microbiome therapeutics, oncolytic viruses, radiotherapy, drugs targeting myeloid-derived suppressor cells, drugs targeting Tregs, drugs targeting renin-angiotensin system, drugs targeting the autonomic nervous system, metformin, and others. These exciting combination therapies hold much promise for cancer patients.

Whereas most ICI research has focused on PD-L1, PD-L2 similarly engages PD-1 and can suppress immune responses. Marinelli et al. evaluated the biological function of PD-L2. They performed in silico analysis of endometrial cancer cell lines using the cancer genome atlas (TCGA) as well as PD-L2 staining of endometrial cancers. PD-L2 was more commonly expressed than PD-L1 in the endometrial cancer cell lines and PD-L2 was also highly expressed in almost 65% of the highly aggressive type II endometrial cancer specimens in both stromal and epithelial components. This alternative ligand for PD-1 appears to have a functional role in these endothelial cancers.

There were two papers that focused on specific immune-related adverse events following ICI therapy. The cardiotoxicity of immune checkpoint inhibitors is discussed by (Chen et al.). Looking at data from the FDA Adverse Event Reporting System (FAERS) database between 2014 and 2019, they noted over 9,000 cases of cardiotoxicity, with males affected close to twice as often as females. Dyspnea, myocarditis, atrial fibrillation, cardiac failure, and pericardial effusion were the top 5 cardiac adverse events reported with myocarditis the only one associated with all the immune checkpoint inhibitors evaluated. Liu et al. discuss the relatively infrequent, but very serious immune-related adverse event of immune thrombocytopenia following ICI treatment. They discuss epidemiology, clinical presentation, and prognosis a well as a case report following treatment with durvalumab. These immune related adverse events highlight the need for careful patient monitoring and the need for additional therapeutic strategies that can avoid some of these adverse events.

In an interesting sociological survey, Zhang et al. performed a national cross-sectional survey of attitudes and practices of ICIs in Chinese patients with cancer. Hesitancy for patients to use ICIs stemmed from high cost, uncertainty about drug efficacy, and no reimbursement from medical insurance. Whereas over 65% of patients reported at least some tumor reduction, a similar proportion reported immune-related adverse events. These findings highlight some of the main problems that limit the use of the current ICIs. The cost/benefit of ICI use was evaluated by Jiang et al. They studied the value assessment of ICI use in terms of the American Society of Clinical Oncology (ASCO) and the European Society for Medical Oncology (ESMO) value assessment frameworks (Jiang et al.). Focusing on nivolumab and pembrolizumab, they noted that of the 19 clinical trials evaluated in various cancer indications, 14 did not meet the ASCO cutoff score while 11 met the ESMO criteria for meaningful value. Interestingly there was only fair correlation between ASCO and ESMO value assessment frameworks.

In a mechanistic paper, Pan et al. studied the effect of dihydropyridine calcium channel blockers in suppressing PD-L1 transcription (Pan et al.). They showed that these agents blocked interferon gamma induced STAT1 phosphorylation thus diminishing PD-L1 expression. This PD-L1 modulation enhanced the killing ability of T cells. This could be a strategy for PD-L1 inhibition therapeutically. PD-L1 modulation was also discussed by (Wang et al.). They studied hepatocellular carcinoma cells (HCC) and found that epidermal growth factor (EGF) stimulation promoted PD-L1 transcription. The EGF stimulation led to PKM2 phosphorylation which in turn led to histone H3 phosphorylation which in turn regulated PD-L1 gene transcription. These findings could provide additional therapeutic strategies in HCC.

The tumor immune microenvironment is important in determining the efficacy of ICI treatment. Wen et al. found that a CTNNA2 mutation changes the immune microenvironment in lung adenocarcinoma patients receiving ICIs. Using lung adenocarcinoma patients in TCGA and a cohort of lung adenocarcinoma patients receiving ICIs, they noted that CTNNA2 mutation was associated with longer OS. The patients with the CTNNA2 mutation had more neoantigens and a greater tumor mutational burden. Furthermore, gene expression levels of CXCL9 and granzyme B were elevated, and the level of the inhibitory receptor killer cell immunoglobulin-like receptor KIR2DL1 was significantly reduced. These findings suggest that CTNNA2 mutation is associated with more immunogenic tumors and this is reflected in the tumor microenvironment.

As noted above, novel therapeutic strategies are needed to both enhance efficacy and limit side-effects of cancer immunotherapies. Numerous novel strategies are proposed in this special issue. Chimeric antigen receptor-modified T cells (CAR-T cells) have been the focus of tremendous interest based on their efficacy in hematologic malignancies. Xiang et al. perform a meta-analysis of prospective clinical trials in patients with refractory/relapsed multiple myeloma treated with CAR-T therapies. They included 27 studies involving 497 patients. The pooled ORR was 89% indicating excellent efficacy in this difficult to treat malignancy. Higher ORR was seen in subgroups of patients including those aged 55 years or less as well as those treated with bispecific CARs targeting both B cell maturation antigen (BCMA) and CD19. They also noted cytokine release syndrome in 76% of patients with 11% grade 3 or higher. This promising approach will certainly be of great interest going forward.

Mohamad Anuar et al. review clinical studies of navitoclax, a BCL-2 family inhibitor (Mohamad Anuar et al.). In phase I and II studies, navitoclax monotherapy potently treats small cell lung cancer and acute lymphocytic leukemia. In combination therapy, it enhances the therapeutic effect of other chemotherapeutic agents in the treatment of solid tumors. It will be interesting to follow the development of this novel agent.

Shi et al. discuss the need for novel immunotherapies (Shi et al.). They note that the tumor immune microenvironment includes exhausted or suppressed T cells, which are the target of the current ICIs. However, other cell types are present which could be taken advantage of. These include dendritic cells, which can activate anti-cancer T cells, neutrophils which may impede the activation and proliferation of T cells, and natural killer cells, which can attack cancer cells lacking MHC class I molecules. Future strategies targeting these cell types are discussed.

In an immunotherapy-related approach, Liu et al. studied patients with oral squamous cell carcinoma (OSCC) and noted higher HER2 expression especially in those with middle and advanced stage cancer. They noted the occurrence of natural autoantibodies to HER2-derived peptides. These antibodies significantly inhibited proliferation and invasion of OSCC cells by inducing the apoptosis and regulated apoptosis-associated factors and epithelial-mesenchymal transition. This kind of bedside to bench research reverses the normal pattern and can provide remarkable insights.

Ubiquitination is an important process regulating the degradation of proteins. This includes immune checkpoint proteins such as PD-L1 and CTLA4. Ubiquitination-mediated protein degradation modulates multiple cellular processes, including transcriptional regulation and cell cycle progression. Deubiquitinating enzymes can prolong protein half-life and thus act as secondary immune checkpoints. Huang et al. review the role of deubiquitinating enzymes in cancer immunity, in particular their direct effects on the stability of pivotal immune checkpoints and other key regulators of T cell function (Huang et al.). These deubiquitinating enzymes may be a target of therapeutic intervention with ICI activity. Ubiquitination is also involved in the activity of a strategy to prevent colitis associated colon cancer development. Patients with ulcerative colitis are at an increased risk for the development of colon cancer. Dai et al. evaluated caffeic acid phenethyl ester (CAPE) as an inhibitor of the NOD-like receptor protein 3 (NLRP3) inflammasome and its efficacy in preventing colitis-associated cancer in a mouse model. They found that CAPE decreased NLRP3 inflammasome activation in bone marrow-derived macrophages and THP-1 cells. CAPE also prevented colon cancer in a murine colitis model. Interestingly, CAPE appeared to work by increasing NLRP3 ubiquitination leading to NLRP3 degradation.

Apolipoprotein A-I (ApoA-I) has anti-inflammatory and anti-oxidative properties. Peng et al. evaluate an Apo-AI mimetic peptide, L-4F, in a mouse model of pancreatic cancer (Peng et al.). They showed that L-4F significantly reduced the tumorigenicity of murine pancreatic cancer cells. In addition, there were effects on myeloid-derived suppressor cells (MDSCs) including apoptosis. Additional effects included differentiation and inhibition of the accumulation of granulocytic myeloid-derived suppressor cells as well as reducing H_2_O_2_ production by MDSCs, increasing T cell proliferation and infiltration into the tumors. Thus, L-4F exerts an anti-tumor and immunomodulatory effect in this murine model of pancreatic cancer by inhibiting PMN-MDSCs.

Zhang et al. discuss paeonol, a phenolic compound with effective anti-inflammatory and anti-tumor properties. They evaluated the effect of paeonol on the mouse lung cancer cell line A549. Paeonol suppressed proliferation and motility of the A549 cells by disrupting STAT3/NF-κB signaling. Paeonol also inhibited the growth of A549 cells transplanted tumors in nude mice. Paeonol could be a candidate for cancer therapy.

The medicinal prospects of algal-derived antioxidants as cancer therapeutics are discussed by (Ferdous and Yusof). They note that cancer therapeutics often induce oxidative damage. Antioxidant supplementation can reduce reactive species levels and mitigate persistent oxidative damage. Their review of the prospective anticancer effect of twenty-three antioxidants from microalgae and their potential mechanism of action, as well as antioxidants from seaweeds, suggests novel agents may be developed both with anti-cancer activity and protective effects against cancer therapeutic side effects.

This special issue benefitted from the tremendous advances being made in cancer immunotherapy. While we have seen great advances, significant challenges remain. Significant side effects from the current generation of ICIs need to be addressed. Also, their limited efficacy in many tumor types. Some of the novel approaches described in this issue hold great promise in ushering in the next generation of cancer immunotherapies.

